# Association Analysis of Polymorphisms in the 5′ Flanking Region of the *HSP70* Gene with Blood Biochemical Parameters of Lactating Holstein Cows under Heat and Cold Stress

**DOI:** 10.3390/ani10112016

**Published:** 2020-11-02

**Authors:** Zaheer Abbas, Lirong Hu, Hao Fang, Abdul Sammad, Ling Kang, Luiz F. Brito, Qing Xu, Yachun Wang

**Affiliations:** 1 Institute of Life Science and Bioengineering, Beijing Jiaotong University, Beijing 100044, China; zaheerabbas@bjtu.edu.cn (Z.A.); 18121612@bjtu.edu.cn (H.F.); lingkang3187@163.com (L.K.); 2 School of Animal Science and Technology, China Agricultural University, Beijing 100193, China; B20193040324@cau.edu.cn (L.H.); drabdulsammad1742@yahoo.com (A.S.); 3 Department of Animal Sciences, Purdue University, West Lafayette, IN 47907, USA; britol@purdue.edu

**Keywords:** heat stress, cold stress, blood biochemical parameters, Chinese Holstein cattle, *HSP70* gene

## Abstract

**Simple Summary:**

Thermal stress causes detrimental effects on the health, welfare, and production of dairy cows, resulting in huge economic losses to the worldwide dairy cattle industry. Understanding the genomic background of thermal stress can lead to more accurate genetic selection strategies. In this study fourteen blood biochemical parameters were evaluated as potential biomarkers for heat or cold stress. Moreover, twelve single nucleotide polymorphisms (SNPs) were detected in the 5′ flanking region of the *HSP70*, a gene known to be associated with thermal stress response in many livestock species. Furthermore, four SNPs were significantly associated with lactate, and lipid peroxide under heat stress, and with dopamine and superoxide dismutase under cold stress. In summary, these molecular markers and bio-markers further our knowledge of thermotolerance in Holstein cattle and can be used for improving breeding strategies for climate resilience.

**Abstract:**

Thermal stress (heat and cold) has large economic and welfare implications for the worldwide dairy industry. Therefore, it is paramount to understand the genetic background of coping mechanism related to thermal stress for the implementation of effective genetic selection schemes in dairy cattle. We performed an association study between 11 single nucleotide polymorphisms having minor allelic frequency (MAF > 0.05) in the *HSP70* gene with blood biochemical parameters. The concentrations of growth hormone (GH), lactate (LA), prolactin (PRL), and superoxide dismutase (SOD) in blood were significantly higher (*p* < 0.05), while the concentrations of blood urea nitrogen (BUN), c-reactive protein (CRP), potassium (K+), lactate dehydrogenase (LDH), lipid peroxide (LPO), and norepinephrine (NE) were significantly lower (*p* < 0.05) in heat-stressed animals as compared to the control group. A significant (*p* < 0.05) increase in the concentrations of cortisol (COR), corticosterone (CORT), and potassium (K+) was observed (*p* < 0.05), while the concentrations of adrenocorticotrophic hormone (ACTH), dopamine (DA), GH, LDH, NE, PRL, and SOD were significantly lower in cold-stressed animals as compared to the control group (*p* < 0.05). Furthermore, SNP A-12G and C181T were significantly associated with LA (*p* < 0.05), while A72G was linked with LPO (*p* < 0.05) in heat-stressed animals. Moreover, the SNPs A-12G and SNP C131G were significantly associated (*p* < 0.05) with DA and SOD under cold stress condition, respectively. These SNPs markers significantly associated with fluctuations in blood biochemical parameters under thermal stress provide a better insight into the genetic mechanisms underlying climatic resilience in Holstein cattle.

## 1. Introduction

Livestock experiences different stressors throughout their lives including physical, nutritional, chemical, psychological, and thermal stress [1,2]. Among them, thermal stress is the most intriguing factor influencing production, reproductive efficiency, health, and the well-being of the high-producing animals [3,4]. In tropical and sub-tropical regions, increased temperature and humidity are the major constraints in livestock production [5], whereas the extremely low temperature in temperate areas also negatively impacts welfare and productivity [1]. The dairy cattle thermo-neutral zone (TNZ) lies in the range of 4 °C to 24 °C, within which the animal maintains a body temperature of 38.4–39.1 °C for normal physiological functions [6]. Temperatures above or below the TNZ threshold can result in heat or cold stress, respectively. The temperature and humidity index (THI) index has been widely used to evaluate thermal stress load in dairy cattle populations [7,8]. The THI threshold for heat stress has been documented to be around 67 [9] and 72 [7,10], and cold stress response is usually activated under THI 38 [11].

Among other effects, thermal stress directly influences feed intake, milk yield, reproductive performance, growth rate and even can cause premature death in harsh conditions [12,13]. Dairy cattle are usually more vulnerable to heat stress because high-producing animals generate more metabolic heat during lactation [5,14,15]. Heat stress is a complex trait as it triggers various physiological and hormonal responses through multiple molecular mechanisms [16]. Moreover, heat stress disturbs the normal regulation of oxidant/anti-oxidants, and thus, causing severe cellular damage through enzymatic and non-enzymatic activities [17,18]. It is also well-known that ambient temperature lower than the TNZ can result in increased feed intake and decreased feed conversion [19]. Temperature-stressed cows also have altered lipids, carbohydrates, and amino acids metabolism [20,21]. Therefore, plasma hormones related to stressor effects are key indicators of the cows’ physiological state and reflect the physiological compensations underlying stress exposure [22]. Various studies have reported that the biological processes involved in complex responses to thermal stress are related to hormonal changes, including insulin, adrenocorticotrophic hormone, dopamine, cortisol, nor-epinephrine, and prolactin concentration [15,21,23]; antioxidants activities, such as superoxide dismutase, glutathione peroxidase; metabolites levels (e.g., blood urea nitrogen, lactate, lipid peroxide) [16,24,25].

Within-breed genetic variation exists for thermo-tolerance and disease resistance [26,27], and thus, genetic and genomic selection for improved heat or cold resistance may increase the resilience and well-being of dairy cattle [2]. Moreover, it will have important implications in the productivity, and long-term sustainability of the dairy industry as the incidence of extreme temperatures is becoming inevitable in the majority of geographic regions around the world [28,29]. *HSP70* is one of the key genes from the heat shock protein (HSP) family, which are associated with thermal stress response [30,31,32,33]. The heat shock protein 70 (HSP70) is involved in many biological activities, including the proper folding of new and abnormal proteins, apoptosis, and cellular immunity [27,33]. Furthermore, HSP70 accomplishes a key role in the normal physiological activities of cells. HSP70 has both inducive and constitutive forms that reduce stress by increasing the chaperone activity [34]. Besides, HSP70 has been shown to regulate protein transport across the cell membrane using energy and hydrolysis, and consequently, maintaining the integrity of the cellular protein [33]. Both in vivo and in vitro studies have shown that HSP70 is also involved in neuro-protection [35].

The 5′ flanking region of the *HSP70* gene is rich in binding sites of various transcription factors, and the mutations in this region can affect the expression of the *HSP70* mRNA, stability, and translation of the HSP70 protein [36,37]. Abundant genetic variation has been reported in the DNA sequence of the *HSP70* gene to discover their link with thermal tolerance capabilities of the animal, the example of which is the investigated variance in the *HSP70* gene associated with heat resistance in Tharparkar cattle [10]. The variation in the promoter region of the *HSP70* gene has been reported to significantly affect the response of fibroblasts to heat stress. Moreover, the mutation in the AP2 locus in the cattle *HSP70* gene results in a significant decrease in the HSP70 transcription level [38]. Basirico et al. found that two mutation sites (g895 C/- and g1128 G/T) in the 5′ UTR region of *HSP70* in Holstein cattle significantly affected the viability of PBMCs and the expression levels of the *HSP70* gene after exposure to heat-stressed condition, the expression level of the *HSP70* gene in mutant individual cells showed significant increase in comparison to the wild type [39]. Polymorphisms in the *HSP70* gene in different sheep breeds raised under thermal stress have shown significant influence (*p* < 0.05) on the blood metabolites such as glucose, serum glutamic-oxaloacetic transaminase (SGOT), phosphorous, triglycerides, and cholesterol [40]. Furthermore, a study conducted by Hu et al. reported significant alteration in four blood metabolites in cattle under cold stress; ACTH (adreno-corticotropic hormone), T3 (tri-iodothyronine), T4 (thyroxine), and K+ (potassium) [27].

Genetic variation in the 5′ flanking region of the *HSP70* gene in Chinese Holstein cattle population and their association with heat or cold stress-related blood biochemical parameters are still unknown. Therefore, this study investigates the naturally occurring fluctuations in blood biochemical parameters during heat and cold stress and its association with the genetic polymorphism in the 5′ flanking region of the *HSP70* gene in Chinese Holstein cattle. The ultimate goal is to identify significant genetic markers and biomarkers that can be used to genetically improve thermo-tolerance in dairy cattle.

## 2. Material and Methods

### 2.1. Animals’ Selection and Sampling

The experiment was performed in agreement with the Committee on Ethics of Animal Experimentation from the Beijing Jiaotong University, Beijing, China (Code ID: SS-QX-2014-06). A total of 196 healthy lactating Holstein cows were selected to evaluate the impact of heat or cold stress on blood biochemical parameters. Out of the total 196 animals, 178 animals were sampled in August 2014 (heat stress period) when THI was higher than 78, 120 in November 2014 (TNZ, THI = 55.43), and 126 in January 2015 (cold stress period) after THI dropped to below 38. Furthermore, rectal temperature was measured three times daily during the three sampling months, and average monthly rectal temperature was calculated then. Most of the animals were repeatedly measured across sampling periods. These animals were raised at the Sanyuan dairy farm in Beijing, China. All the animals were maintained under general management practices, fed in a scattered hurdle with a full mixed ration three times a day and unrestricted access to fresh drinking water. Experimental cows were kept in a semi-open barn, with sprinklers above the feeding line and fans over the stall space. In the summer, cooling sessions were performed during feeding periods. Free-stall fans and sprinklers were operational during the entire month of August (hottest period in the area). During night time, cows were allowed to rest in the outer-barn free yard, according to the weather conditions. Milking was carried out three times a day (morning, afternoon, and evening). In August cows were subjected to a rigorous cooling session of 30 min prior to milking.

Blood samples were collected from the coccygeal vein into anticoagulant-mixed and anticoagulant-free tubes. The anticoagulant-free blood samples were centrifuged at 3000 rpm/min for 10 min. The upper serum layer was parted and frozen at −80 °C for the determination of blood biochemical parameters. Furthermore, the anticoagulant-mixed blood was used to extract genomic DNA according to the kit instruction (DP318-02, TIANGEN Biotech—Beijing, Co. Ltd., Beijing, China).

### 2.2. THI Calculation

The temperature and relative humidity in percentage (RH) were measured using an automatic weather station (Model: P4581, Comet System S.R.O., Bezrucova, Czech Republic) located within the lactating-cows’ pen. THI was recorded from 1 August (summer), where the THI was higher than 78 continuously for 7 days, while THI for the TNZ condition was recorded after 1 November and for cold stress after 1 January. Furthermore, THI was calculated as [41]:

THI = 0.8 × AT + (RH (%)/100) × [(AT − 14.4) + 46.4](1)

where AT is the air temperature in Celsius degree (°C) and RH denotes the percentage (%) relative humidity.

### 2.3. Detection of Blood Biochemical Parameters

Fourteen blood metabolites were evaluated as potential indicators of heat or cold stress response, including ACTH (adrenocorticotrophic hormone), COR (cortisol), CORT (cortisone), CRP (c-reactive protein), DA (dopamine), GH (growth hormone), LPO (lipid peroxide), NE (nor-epinephrine), PRL (prolactin), and SOD (superoxide dismutase), which were measured using radioimmune-assay [42]. Moreover, BUN (blood urea nitrogen), LA (lactate), and LDH (lactate de-hydrogenase) were detected by the colorimetry technique [43], while K+ (potassium) was measured through an electrolyte analyzer. The laboratorial work was done at the Beijing Huaying Biotechnology Research Institute, Beijing, China.

### 2.4. PCR Amplification and Sequencing

The isolation of genomic DNA from the blood samples was performed according to the manufacturer instruction (DP318-02, TIANGEN Biotech Co., Ltd., Beijing, China). The primer for the targeted region (5′ flanking area) of the *HSP70* gene was constructed based on DNA sequence AY149618.1 (GenBank accession number) using the Primer version 3.0 (PREMIER Biosoft, San Diego, CA, USA). The forward primer was 5′GTCGTGTAGCCCTTAATTCTA3′, and the reverse primer was 5′ACGCAGGAGTAGTGGTG3′. The amplification system of PCR was 50 μL, including 2 μL bovine genomic DNA (50 ng/μL), 5 μL 10× PCR Buffer, 4 μL dNTP Mixture (2.5 mM), 2 μL forward primer (10 μM), 2 μL reverse primer (10 μM), and 0.25 μL rTaq enzyme (5 U/μL). The reaction conditions were 94 °C 5 min; 94 °C 45 s; 58 °C 30 s; 72 °C 60 s; 30 cycles; and 72 °C for 5 min. The purification of the PCR product was done using the Gel Extraction Kit (Omega Bio-Tek, Norcross, GA, USA). Furthermore, the amplified fragments were sequenced both in forward and reverse directions using BigDye^®^ Terminator v3.1 Cycle Sequencing Kit (Thermo Fisher, South San Francisco, CA, USA).

### 2.5. Detection of SNPs in the 5′ Flanking Region of the HSP70 Gene

The PCR amplification products were sent to the Bioengineering Co. Ltd. laboratory (Shanghai, China) for sequencing. Single nucleotide polymorphisms (SNPs) located in the 5′ flanking region of the *HSP70* gene were detected by analyzing the polymorphic loci of the target sequence in the DNA samples of 196 Holstein cows using the DNAMAN version 6.0 (Lynnon Biosoft, San Ramon, CA, USA) and Chromas2.0 (Technelysium, South Brisbane, Australia) software.

### 2.6. Association Analyses

The MIXED procedure implemented in SAS9.2 software (SAS Institute Inc., Cary, NC, USA) was applied to estimate the effect of heat or cold stress on blood metabolites of Holstein cows. The statistical model for the effect of heat or cold stress on blood metabolites is described as:

y = μ + SEA + PAR + Cow + b1 × DIM + b2 × DIM2 + e(2)

where “y” is the individual blood biochemical content measured in January, August, or November; “μ” is the mean of each blood biochemical content; “SEA” is the fixed effect of season; “PAR” is the fixed effect of parity; “Cow” is the individual effect of the animal; “DIM” is the effect of days in milk; “b1” and “b2” are the regression coefficients and “e” is the residual term.

Subsequently, association analyses between the *HSP70* gene polymorphisms and blood metabolites affected by heat or cold stress were performed using the GLM procedure of SAS 9.2 (SAS Institute Inc., Cary, NC, USA). The model is presented as:

y = μ + SNP + PAR + b1 × DIM + b2 × DIM2 + e(3)

where “y” is the individual blood biochemical content measured in August or January; “μ” is the average of each blood biochemical content; “SNP” is the fixed effect of different genotypes of each SNP; “PAR” is the fixed effect of parity; “DIM” is the effect of days in milk; “b1” and “b2” are the regression coefficients, and “e” is the residual term. The significance threshold used was *p* < 0.05.

## 3. Results

### 3.1. Air Temperature, THI, and Rectal Temperature during Thermal Stress and TNZ Period

The average temperature and THI in the experimental cowshed were monitored for three months as shown in Table 1. The threshold categories set for thermal stress are mild, moderate, and extreme [44]. The currently reported categories set for THI are: 68 ≤ THI < 72, is considered to cause mild heat stress, 73 ≤ THI < 79 represents moderate heat stress, and 80 ≤ THI < 89 indicates, while THI ≥ 90 is considered as fatal/emergency state [45]. In Beijing, the average temperature and THI of the cows-shed in August/2014 was 31.80 ± 2.80 °C and 81.57 ± 3.20, respectively, and the daily average THI greater than 78 was observed for 21 days in August/2014, which lasted for 8 h. This indicates that the experimental animals were under severe heat stress (72.40 < THI < 89.68). The average temperature and THI in November/2014 were 12.76 ± 3.92 °C and 55.43 ± 5.43, respectively, showing that the cows were under TNZ conditions. The average temperature in January/2015 was −6.70 ± 2.35 °C and the average THI was 25.63 ± 4.67 revealing that the cows were under mild cold stress. The animals’ rectal temperatures were recorded three times daily during the three months of sampling, and the average monthly rectal temperature was calculated. The rectal temperature in August (summer) showed a significant increase as shown in Table 1, indicating that an external increase in temperature and THI has a positive effect on the internal body temperature.

### 3.2. Heat or Cold Stress Effect on Blood Biochemical Parameters of Holstein Cows

Analysis of variance shows that season, parity, and individual cows had a significant effect on a variety of blood biochemical parameters (*p* < 0.05). The parity and individual effects were adjusted through the least square method and season comparison was performed as shown in Table 2. The blood concentration of GH, LA, PRL, and SOD under heat stress condition increased significantly (*p* < 0.05) as compared to the TNZ condition, while there was a significant decrease (*p* < 0.05) in BUN, CRP, K^+^, LDH, LPO, and NE. Furthermore, the concentration of COR, CORT, and K^+^ was higher under cold stress conditions compared to TNZ (*p* < 0.05), while the concentrations of ACTH, DA, GH, LDH, NE, PRL, and SOD were significantly lower (*p* < 0.05) under cold stress. The significant changes in blood metabolites in response to thermal stress indicate that they are potential biomarkers for thermo-tolerance.

### 3.3. SNPs in the 5′ Flanking Region of the HSP70 Gene

**Figure 1:**
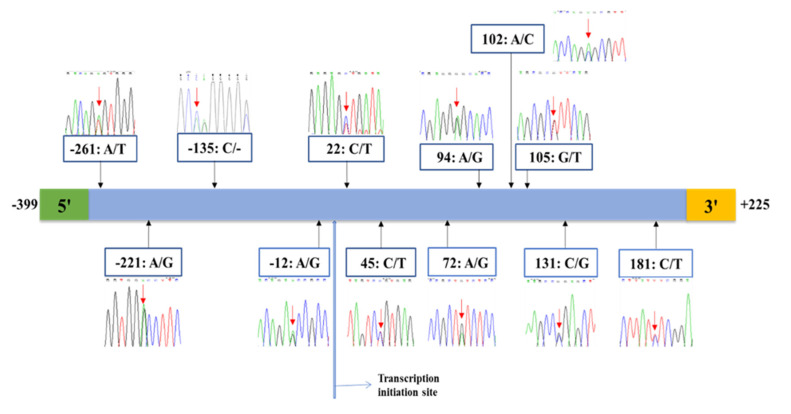
Diagrammatic representation of the detected SNPs within the 5′ flanking region of the *HSP70* gene in Chinese Holstein cattle. The nucleotide position is according to the sequence alignment of Holstein Friesian cattle (AY149618.1).

The 5′ flanking region of the *HSP70* gene was amplified and sequenced in 196 Holstein cows. Based on the reference sequence of Holstein Friesian (AY149618.1), the amplified product was 624 bp including 399 bp of the 5′ flanking region, 208 bp of the untranslated region (5′ UTR), 17 bp of the coding region, and 225 bp of the first exon. The target region was then sequenced and analyzed further for SNP detection. The SNPs observed in the 5′ flanking region of the *HSP70* gene in Holstein cows are shown in Figure 1. Total of 12 SNPs were identified, in which all these SNPs were reported in our previous study except SNP A-221G, and A-12G [27]. Six out of the 12 SNPs (A-261T, A-221G, C-135-, A22T, G105T, and C131G) were not found in the dbSNP database (www.ncbi.nlm.nih.gov). Out of these six SNPs, three SNPs (A-261T, A-221G, and C131G) did not have the information on chromosomal position. The gene and chromosomal location, accession number, and allelic frequency information of the 12 SNPs are presented in Table 3.

### 3.4. Association Analyses of SNPs and Blood Biochemical Parameters Related to Thermal Stress

Association analyses were performed between 11 SNPs with a MAF greater than 0.05 (Table 3) and the blood biochemical parameters significantly related to thermal stress response in cattle (Table 2). Four SNPs (A-12G, A72G, C131G, and C181T) detected in the 5′ flanking region of the *HSP70* gene were significantly associated with blood biochemical parameters (LA, DA, LPO, and SOD) in Holstein cows under heat or cold stress (Table 4). Under heat stress, SNP 4 (A-12G) and 12 (C181T) were significantly associated with LA content in blood (*p* < 0.05). The Bonferroni *t*-test indicates that the LA content in individuals with the GG genotype of the SNP A-12G was significantly higher compared to the AG genotype, while the individuals with TT genotype of SNP C181T had a higher LA concentration under heat stress conditions. Furthermore, SNP 7 A72G was significantly associated with LPO content (*p* < 0.05), in which the LPO content in individuals with AA and AG genotypes was significantly higher compared to the GG genotype. Moreover, the polymorphism located within the 5′ flanking area of the *HSP70* gene was also significantly associated with the blood biochemical parameters under cold stress (Table 4). For instance, SNP 4 (A-12G) was significantly correlated with DA content (*p* < 0.05), in which AA genotype individuals had significantly higher DA concentration compared to AG and GG genotypes. SNP 11 (C131G) was also associated with SOD activity (*p* < 0.05), in which SOD activity was significantly higher (*p* < 0.05) in individuals with GG genotype compared to CC and CG genotypes.

## 4. Discussion

**Figure 2:**
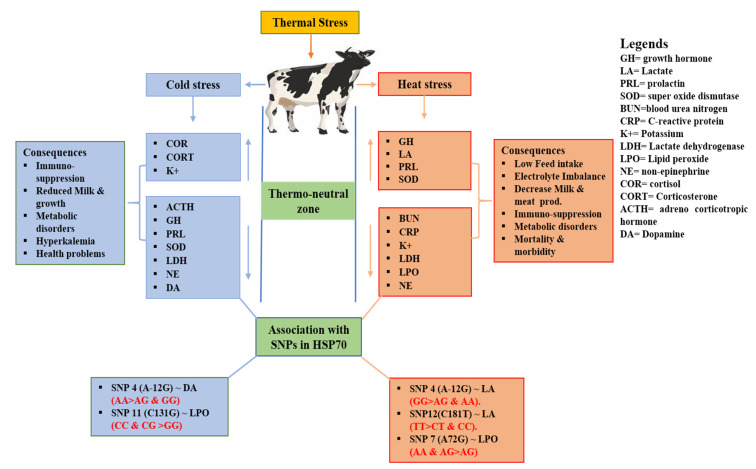
Schematic representation of the blood biochemical parameters changes under heat and cold stress, and its consequences along with the association between significant blood biochemical parameters and SNPs detected in the 5′ flanking region of the *HSP70* gene in Chinese Holstein cattle.

THI is widely used to quantify thermal stress in dairy animals [29,46]. When THI exceeds 72, the animals experience heat stress [12,29], and cold stress for THI lower than 38 [11]. In the present study, the average THI exceeded 72, showing that animals were under heat stress in August/2014 and cold stress in January/2015 when the average THI was lower than 38. The rectal temperature recorded during the summer significantly increased, while no significant changes in the rectal temperature was observed during the winter period, as shown in the Table 1. Thermal stress causes physiological, hormonal, biochemical, and molecular responses for the sake of cell survivability in harsh and stressful environments [15,16,21]. Our findings show that there were significant changes in blood metabolites of Holstein cattle under heat or cold stress as shown in Figure 2, in agreement with other studies in Holstein and Jersey cattle [47,48].

In our study, the concentration of PRL, GH, LA, and SOD significantly increased while BUN, CRP, K+, LDH, LPO, and NE decreased in heat-stressed cows compared to the TNZ. The animal body increases heat dissipation under heat stress, which manifests as shortness of breath, rapid heartbeat, and disorder of metabolic pathways [5]. The change in PRL has a direct relationship with increased rectal temperature [49], which is consistent with our study. PRL is not only correlated with milk production but also involved in thermoregulation by maintaining water and electrolytes balance [50]. The increase in PRL is to meet up with the increased demand for water and electrolytes during heat stress [51]. GH establishes a prominent position along with PRL in milk synthesis, as well as partitioning of energy toward the mammary gland for milk synthesis during heat stress [52]. The animal experiences negative energy balance during heat stress because of reduced feed intake [21]. The possible reason for increased GH is to enable maintenance of the energetic status of the animal during heat stress condition [52]. LA increased during heat stress in the leg and pectoral muscles of chickens [53], as well as in the blood of Holstein cattle [54], which corroborates with our findings. Heat-stressed cows showed increased gluconeogenesis as compared to TNZ conditions [55], and presumably increased utilization of LA and alanine for glucose synthesis [21,55,56]. Besides the animals dissipating heat through panting and sweating during heat stress that results in dehydration [57], the increase in LA may also be due to decreased blood volume as a result of dehydration and lower blood flow toward the muscle during heat stress [58]. Heat stress induces oxidative stress that stimulates certain body defense mechanisms and increase oxidative stress-related biomarkers such as SOD in order to scavenge free radicals [59]. Various studies claimed that heat stress increases the blood SOD in cows [60], goats [61], and mice [62], which is similar to our findings.

BUN is normally originated from rumen and through de-amination of the amino acids by the liver [63]. Srikandakumar et al. reported that the BUN content in dairy cattle decreased during heat stress by 1.48 mmol/L and 0.65 mmol/L, respectively, which is consistent with the results of this study [64]. The decrease in BUN concentration suggests an alteration in protein catabolism, and the nitrogenous re-partition during heat stress [15]. Moreover, heat stress elevates the marginal vasodilation to dissipate more heat resulting in decreased blood drift to the organs [15], besides the increased dehydration status reducing blood flow to the kidneys. The decrease in BUN during heat stress may also be due to the malfunctioning of the kidneys during heat stress [65]. CRP is known as a sensitive inflammation marker, the decrease in CRP may be due to liver dysfunction due to heat stress, as CRP is liver originated during diseases or under severe stress. Another study claimed that CRP is correlated with milk production and is at peak during high lactation [66], thus the decrease of CRP in heat-stressed cows may also be as a result of decreased milk production due to reduced feed intake during heat stress. LPO decreased during heat stress conditions, likely as defensive capability of HSP70 [67], and the increased activity of the antioxidant defense system against per-oxides, which includes various enzymes such as SOD, catalase (CAT), and glutathione per-oxidase during heat stress [68,69,70]. Previous studies have shown that LDH concentration is lower in the summer in comparison to the TNZ, as also noticed in the current study, and is thought to be the consequence of the reduced thyroid activity during heat stress [71,72].

The main physiological response of animals under cold stress is to up-surge heat production and decrease heat dissipation in order to up-hold a constant body temperature, besides it also changes the endocrine hormone regulation [73]. Similar to our study, the DA level in chickens decreased after exposure to severe cold stress [74], which may be due to the damage of neural spikes of DA neuron releasing DA. In our study, the COR and CORT plasma levels were higher under cold stress, while different studies showed a similar trend in the increase of blood COR in pigs and dairy cattle under cold stress [19,75]. Moreover, a recent study showed that blood COR increased in dairy cattle after the exposure to heat stress for a long duration [76], likely to reduce metabolic heat production [11]. Similar to heat stress, in order to maintain body temperature and elevate energy production, cows under cold stress can reduce production performance and immunity [77]. Based on the above evidences, the increase in COR may be an adaptive strategy and reflect a shielding action favoring an increase in metabolic heat production under cold stress. Furthermore, cold stress was also found to increase corticosterone and thyroxin levels. In summary, heat or cold stress caused significant changes in blood metabolites in Holstein cows, which had a great impact on the physiological state of the cows and reflect the physiological compensations that cows undergo at various lactation intensities and/or stress exposure. Therefore, the aforementioned metabolites can be used as bio-markers for evaluating tolerance to thermal stress.

The HSP70 protein family not only acts as a molecular chaperone to assist the folding/unfolding, congregation, and transportation of new and abnormal proteins, inhibit cell apoptosis, and protect cells from stress damage, but also stimulate the rapid response of the immune system and regulate the activation of immune cells and the production of cytokines [78,79]. SNPs provide tools to carry out association analyses and the identification of genetic markers for the selection of important traits [27,80]. It has been reported that SNPs in the 5′ flanking region of the *HSP70* gene in Duroc boars show association with semen quality under heat stress, and suggested that those SNPs can contribute as genetic markers for breeding for improved heat tolerance in pigs [81]. Polymorphisms in the 5′ flanking regions of the *HSP70* gene validate its association with the blood metabolites such as T3 and T4 in Sanhe cattle under severe cold stress [27]. Deb et al. reported that the variation of the AP2 binding element in the promoter region of the *HSP70* gene could affect heat stress response of Frieswal hybrid cattle [82]. The rectal temperature and respiratory frequency of homozygous genotype individuals were significantly lower (*p* < 0.05) than those of heterozygous individuals. We identified a total of twelve SNPs in the 5′ flanking region of the *HSP70* gene (Table 4). All these SNPs were previously reported under cold stress by our group except SNP A-221G, and A-12G [27]. However, the inclusion of three distinct weather conditions with respective representation of important biochemical parameters in high producing cows, constitute salient features of this association study. We found that four SNPs were significantly associated with the various blood biochemical parameters (Table 3) related to heat or cold stress in Chinese Holstein cows. Under heat stress three SNPs (A-12G, C181T, and A72G) were significantly associated with the blood metabolites LA, and LPO. Under cold stress SNPs, A-12G and C131G were significantly associated with DA and SOD, respectively. The validation of these blood biomarkers under heat stress and their association with the polymorphisms in the 5′ flanking region suggest them as indicators of thermal stress in Holstein cattle. Furthermore, the polymorphisms in the 5′ flanking region associated with these biochemical indicators reveal that three SNPs (A-12G, A72G, C181T) may be used as molecular marker for the selection of heat resilient animals, while A-12G and C131G may be used as biomarkers for cold tolerance. Future studies should perform associations with whole-genome markers to identify other SNPs associated with the biomarkers defined here.

## 5. Conclusions

This study has taken a novel approach for the characterization of blood biochemical profile of lactating Holstein cows under three thermal conditions. The association analysis performed hereafter identified SNPs in the 5′ flanking region of the *HSP70* gene, which are significantly associated with the indicators of thermal tolerance. An antagonistic trend of gluconeogenesis-related biochemical parameters between cold and heat stress depicts the cow’s physiological alterations to thermal challenges. SNP A-12G and C181T were significantly associated with lactate; SNP A72G with lipid per-oxide under heat stress; while SNP A-12G was significantly associated with dopamine; and SNP C131G was linked with superoxide dismutase under cold stress. Moreover, these SNPs may be used as candidate molecular markers for thermo-tolerance in Chinese Holstein cattle.

## Figures and Tables

**Table 1:** Air temperature, temperature and humidity index (THI), and rectal temperature calculated during three different seasons.

Parameter	August/2014	November/2014	January/2015
Temperature (°C)	31.80 ± 2.80	12.76 ± 3.92	−6.70 ± 2.35
Min~Max Temperature (°C)	22.87–36.35	4.86–19.91	−9.89–−1.90
THI	81.57 ± 3.20	55.43 ± 5.43	25.63 ± 4.67
Min~Max THI	72.40–89.68	40.84–63.92	18.10–36.69
Rectal temperature	38.68 ± 0.03 ^a^	38.53 ± 0.03 ^b^	38.51 ± 0.03 ^b^

Note: ^a,b^ Different letters in the last row indicate significant differences between the periods (*p* < 0.01).

**Table 2:** Changes in the blood biochemical parameters under heat or cold stress.

Blood Parameters	Heat Stress (LSM ± SE) ^1^	TNZ (LSM ± SE) ^1^	Cold Stress (LSM ± SE) ^1^	*p*-Value ^2^
ACTH (pg/mL)	23.26 ± 0.51 ^ab^	24.08 ± 0.62 ^a^	21.43 ± 0.63 ^b^	0.008
BUN (mmol/L)	5.00 ± 0.07 ^b^	5.59 ± 0.09 ^a^	5.39 ± 0.09 ^a^	<0.0001
COR (ng/mL)	95.66 ± 1.38 ^b^	95.07 ± 1.67 ^b^	104.20 ± 1.69 ^a^	<0.0001
CORT (ng/mL)	286.64 ± 0.92 ^b^	286.17 ± 1.12 ^b^	292.90 ± 1.13 ^a^	<0.0001
CRP (mg/L)	2.99 ± 0.08 ^b^	4.40 ± 0.09 ^a^	4.18 ± 0.09 ^a^	<0.0001
DA (ng/mL)	73.28 ± 1.89 ^ab^	78.12 ± 2.28 ^a^	67.22 ± 2.32 ^b^	0.0027
GH (ng/mL)	5.26 ± 0.05 ^a^	3.95 ± 0.07 ^b^	3.41 ± 0.07 ^c^	<0.0001
K+ (mmol/L)	14.49 ± 0.13 ^c^	16.44 ± 0.16 ^b^	17.43 ± 0.16 ^a^	<0.0001
LA (mmol/L)	2.22 ± 0.02 ^b^	2.05 ± 0.03 ^a^	2.06 ± 0.03 ^a^	<0.0001
LDH (U/L)	901.82 ± 17.47 ^b^	1008.61 ± 21.03 ^a^	951.36 ± 21.40 ^b^	<0.0004
LPO (nmol/L)	5.49 ± 0.04 ^b^	5.80 ± 0.05 ^a^	5.74 ± 0.05 ^a^	<0.0001
NE (pg/mL)	355.34 ± 5.53 ^b^	425.06 ± 6.72 ^a^	408.18 ± 6.78 ^b^	<0.0001
PRL (uIU/L)	292.64 ± 2.69 ^a^	221.04 ± 3.27 ^b^	209.26 ± 3.30 ^c^	<0.0001
SOD (U/mL)	120.28 ± 0.92 ^a^	111.64 ± 1.12 ^b^	93.54 ± 1.13 ^c^	<0.0001

^1^ Multiple comparisons were performed by the Bonferroni *t*-test after adjusted for the significant fixed effects. LSM, least-square means; SE, standard error. ^a,b,c^ Different letters in the same row indicate significant differences between the two months (*p* < 0.05). ^2^ *p* < 0.01 indicates highly significant differences among seasons; (*p* < 0.05) indicates a significant difference among seasons.

**Table 3:** SNPs detected in the 5′ flanking region of the *HSP70* gene in Chinese Holstein cows.

SNP	Gene Position ^1^	Chromosome Position	Accession Number	Allele Frequency ^2^	
1	−261	NA	NA	A:0.8053	T:0.1947
2	−221	NA	NA	A:0.0053	G:0.9947
3	−135	23:27,334,006	NA	C:0.7193	−:0.2807
4	−12	23:27,333,887	rs445536803	A:0.1754	G:0.8246
5	+22	23:27,333,854	NA	A:0.9474	T:0.0526
6	+45	23:27,333,831	rs211506802	A:0.7965	T:0.2035
7	+72	23:27,333,804	rs471604061	A:0.9263	G:0.0737
8	+94	23:27,333,782	rs438646103	A:0.1860	G:0.8140
9	+102	23:27,333,774	rs478612967	A:0.4158	C:0.5842
10	+105	23:27,333,771	NA	G:0.6456	T:0.3544
11	+131	NA	NA	C:0.9421	G:0.0579
12	+181	23:27,333,726	rs473916108	C:0.5895	T:0.4105

Note: ^1^ The transcription initiation site is +1; ^2^ allele frequency of each SNP in the 196 individuals used in this study.

**Table 4:** Significantly associated SNPs in the 5′ flanking region of the *HSP70* gene of Holstein cows with the blood metabolites under thermal stress.

SNPs	Stress	Blood Biochemical Parameters	*p*-Value ^1^	Genotype	Number of Animals	Least Squares Mean + Standard Error ^2^
4 (A-12G)	Heat stress	LA (mmol/L)	0.03	AA	13	2.16 ± 0.12 ^ab^
				AG	33	2.06 ± 0.08 ^b^
				GG	124	2.27 ± 0.05 ^a^
	Cold stress	DA (ng/mL)	0.03	AA	9	84.66 ± 7.44 ^a^
				AG	26	62.08 ± 4.68 ^b^
				GG	86	65.74 ± 3.02 ^b^
7 (A72G)	Heat stress	LPO (nmol/mL)	0.02	AA	152	5.51 ± 0.06 ^a^
				AG	14	5.80 ± 0.15 ^a^
				GG	4	4.72 ± 0.36 ^b^
11 (C131G)	Cold stress	SOD (U/mL)	0.04	CC	110	96.47 ± 1.21 ^b^
				CG	10	94.60 ± 3.11 ^ab^
				GG	1	113.12 ± 6.42 ^a^
12 (C181T)	Heat stress	LA (mmol/L)	0.05	CC	81	2.16 ± 0.15 ^b^
				CT	28	2.19 ± 0.08 ^ab^
				TT	61	2.34 ± 0.06 ^a^

Note: ^1^ *p* < 0.05 indicates significantly associated with the blood biochemical parameters. ^2 a,b^ Different letters in the same column indicate a significant difference between genotypes (*p* < 0.05) based on the Bonferroni *t*-test. The association between all the 11 SNPs and blood biochemical parameters was done, but only 4 SNPs were found significantly associated with the above blood biochemical parameters.
